# Safety, clinical and laboratory characteristics of donors with thalassemia minor in living donor kidney transplant: a case series

**DOI:** 10.1186/s12882-021-02609-2

**Published:** 2021-12-01

**Authors:** Nhan Hieu Dinh, Suzanne Monivong Cheanh Beaupha

**Affiliations:** 1 Department of Internal Medicine, University of Medicine and Pharmacy at Ho Chi Minh City, Ho Chi Minh City, Vietnam; 2 Department of Pharmacology, University of Medicine and Pharmacy at Ho Chi Minh City, Ho Chi Minh City, Vietnam; 3 Department of Hematology, University of Medicine and Pharmacy at Ho Chi Minh City, Ho Chi Minh City, Vietnam

**Keywords:** Thalassemia, Living donor kidney transplant, Safety, Donors

## Abstract

**Background:**

Due to the increasing demand for kidney transplants, sometimes donors with underlying medical conditions can be considered for living kidney donor transplant. Thalassemia is amongst the most common inherited disorders of hemoglobin globally, which is not restricted as an exclusion criterion. However, there is currently no study examine the safety and characteristics of kidney donors with thalassemia minor.

**Methods:**

All eligible live kidney donors between 2016 and 2019 with thalassemia minor at a tertiary hospital were recruited. Baseline characteristics, clinical and laboratory outcomes were investigated.

**Results:**

Fifteen donors (11 women, 55.5 ± 15.0 year-old) were included with a follow-up duration of 2 (1-4) years since operation. The most prevalent gene mutation among participants was DEL-SEA. No clinical manifestations of anemia were seen but 10 participants had mild anemia diagnosed from blood tests. Cardiovascular, liver and renal function were normal before nephrectomy. Until now, all donors are alive and maintain overall good health. Anemia condition is not affected, and the post-donation eGFR = 71.04 ± 11.54 mL/min/1.73m^2^ is comparable to outcomes of healthy donors reported in previous studies. Two donors are at risk of proteinuria at 1-year post-transplant with A/C ratio > 30 mg/g.

**Conclusions:**

Thalassemia minor individuals who are non-transfusion-dependent, without anemia clinical manifestations and have no contraindications to kidney donation are safe to be donors in short-term. An eGFR of at least 80 mL/min/1.73m^2^ should be considered to avoid low post-donation eGFR, and awareness should be raised on thalassemia donors with even mild albuminuria. Nephrectomy does not worsen thalassemia.

**Supplementary Information:**

The online version contains supplementary material available at 10.1186/s12882-021-02609-2.

## Background

For patients with end-stage renal disease, living donor kidney transplant is considered as a preferred treatment that confers the best long-term outcomes for eligible recipients compared to other available options including deceased donor kidney transplantation and dialysis [1]. In several countries such as the United States and Taiwan, the number of patients waiting for kidney transplantation is rapidly increased recently and accounted for the highest proportion among organ transplantation indicates a high demand for kidney transplantation of which the current number of donors is insufficient to meet the existing demand [2, 3]. The donors must meet the criteria of an authorized organ donor to not only ensure the quality of the kidney which will improve the prognosis of kidney transplant for recipients but also minimize the impact on the donor’s health post-surgery. Kidney donation criteria have been set and update periodically to achieve the best results for the kidney transplant process [4].

Due to the increasing demand for kidney transplants worldwide and particularly in Vietnam, in specific cases, donors who have underlying medical conditions with minimal effect on health and prognosis after kidney donation can also be considered. Thalassemia is amongst the most common inherited disorders of hemoglobin globally with a prevalence of 8.5% α-thalassemia and 2.5% β-thalassemia among the general population in a study conducted in China [5]. Donors with thalassemia minor who do not require blood transfusions are not restricted as exclusion criteria for living donor kidney transplant. However, it is proposed that several abnormalities in renal tubular function were found among thalassemia minor patients [6]. Mechanisms of renal manifestations in thalassemia minor involve chronic anemia, iron overload and iron chelation therapy [7], of which low-grade hemolysis, tubular iron deposition and erythrocytes-derived toxins might be considered as specific causes associated with thalassemia minor [6]. Kidney transplant with living thalassemia minor donors therefore require more attention and close monitoring before and after transplantation.

There is currently no study investigating donors with thalassemia minor in living donor kidney transplantation. This study aimed to investigate the safety, clinical and laboratory characteristics of donors with thalassemia minor in living donor kidney transplant, and examine characteristics that need to be taken into account in these specific individuals.

## Methods

A retrospective study was conducted in Cho Ray Hospital, Vietnam from November 2020 to April 2021. Eligible live kidney donors between 2016 and 2019 with thalassemia minor confirmed by hemoglobin electrophoresis and/or genetic analysis were recruited. According to medical criteria that are acceptable for kidney donation, except thalassemia minor, participants were healthy individuals aged between 18 and 70 year-old without chronic diseases such as diabetes, coronary artery disease, infectious disease, or cancer. Flexible criteria are considered when the patients have difficulty finding a suitable compatible donor, such as the donor can have mild chronic disease which had been under well-managed medical control. Clinical and laboratory characteristics of participants during their follow-up between November 2020 and April 2021 were recorded and compared to their corresponding results pre-donation.

The donors were scheduled to visit the clinic at 1 month, 3 months, 6 months post-surgery. If their conditions are stable, the patients will be followed up annually until death or withdrawal. If the donor does not arrive to their appointment, phone calls will be made to ensure that they are still alive and reschedule to a new date. At each follow-up visit, we screened for post-surgery complications including infection (surgical site, urinary tract, respiratory tract, gastrointestinal infection), haemorrhage, anemia, hypertension, angina, proteinuria, decreased renal function. The latest data of their visit is collected. Estimated glomerular filtration rate (eGFR) values were calculated using the CKD-EPI formula.

The analyses and results interpretation were reviewed by a biostatistician. To demonstrate baseline variables, continuous data were presented by mean values and standard deviation which appropriate to the normality of data, while categorical data were described using frequencies and percentages. Pre-surgery and post-surgery data were compared with paired t-test or paired Wilcoxon test appropriately. Ethical approval was obtained from the local institutional review board and consent for publication was obtained from all donors.

## Results

A total of 15 donors with thalassemia minor were admitted to our hospital during the study period. The donors were followed up for a median duration of 2 (1 – 4) years. Until now, all donors are alive and maintain overall good health. The majority of donors are female who are first-degree relatives of the recipients and have α-thalassemia. The most prevalent gene mutation among participants was DEL-SEA, which occurred in 12 out of 15 participants (Table 1).

**Table 1:** Baseline characteristics of participants

Characteristics	
Age	55.5 ± 15.0 (45.0 – 70.0)
Sex	
Female	11/15 (73.3%)
Male	4/15 (26.7%)
Relationship to recipients	
Bloodline relatives (parent, sibling)	14/15 (93.3%)
Spouse	1/15 (6.7%)
Type of thalassemia	
α-thalassemia	13/15 (86.6%)
β-thalassemia	1/15 (6.7%)
Hemoglobinose	1/15 (6.7%)
Gene mutation	
DEL-SEA	12/15 (80.0%)
c.-78 A > G of HbB gene	1/15 (6.7%)
Unknown	2/15 (13.3%)
Duration of follow-up	2 (1 – 4) years, min: 4 months, max: 5 years

Table 2 shows the comparison between pre-surgery laboratory characteristics and their latest data. Before kidney transplant, all donors showed no clinical manifestations of anemia and no enlarged spleen, however 10 participants had mild anemia diagnosed from blood test. No abnormal cardiovascular signs have been reported, the donors had mean blood pressure <140/90mmHg and average EF = 68 ± 4.66%, except 2 donors with mild hypertension who had been well-managed with medically controlled until present. Liver functions are normal. The shape and size of 2 kidneys and renal arteries were normal according to results from ultrasound and CT scan. Renal function were normal considering creatinine, eGFR and renal scintigraphy results. The latest follow-up examination (4 months to 5 years post-surgery) showed no difference in anemia condition among the donors. Renal function decreased with eGFR = 71.04 ± 11.54 compared to 94.50 ± 5.39 pre-surgery (p < 0.001). An increase of eGFR from 92 to 101.06 mL/min/1.73m^2^ was observed in Donor 1 after 28 months of follow-up.

In general, there is no significant difference between A/C ratio values pre- and post-surgery (p = 0.322), but two donors had A/C ratio values increased at an alarming rate which indicates possibilities of kidney damage occur post kidney transplant (Supplementary Table 1). Serum potassium, sodium, calcium levels, rapid urine test results were within normal range. Most donors had no post-surgery complications, except the two donors were at risk of proteinuria. The pre-surgery A/C ratio value of Donor 10 was 8.05 mg/g, after one year of follow-up it increased to 173 mg/g in November 2020. Donor 13 who had the surgery in December 2018 had a pre-surgery A/C ratio of 14.7 mg/g, which remained normal (13.1 mg/g) on her first follow-up visit in January 2019 but increased to 34.2 mg/g on her latest visit in December 2020. Both donors do not have any clinical symptoms or comorbidities that can affect kidney function.

**Table 2:** Clinical and laboratory characteristics of participants

Characteristics	Before kidney transplant	After kidney transplant	p-value
Hb (g/L)	117.64 ± 16.50	117.07 ± 13.54	0.842
Hct (%)	38.57 ± 3.88	38.12 ± 3.56	0.673
Anemia	10/15 (66.6%) Hb (anemia): 108.76 ± 12.50	10/15 (66.6%) Hb (anemia): 110.36 ± 8.14	0.735
Non-anemia	5/15 (33.3%) Hb (non-amenia): 135.40 ± 3.21	5/15 (33.3%) Hb (non-amenia): 135.50 ± 4.12	0.969
Systolic blood pressure (mmHg)	123.67 ± 12.88	119.33 ± 8.84	0.144
Diastolic blood pressure (mmHg)	74.40 ± 8.89	74.00 ± 5.07	0.839
Heart rate	78.00 ± 6.23	77.73 ± 5.70	0.874
ECG	Normal		
EF on echocardiogram (%)	68.00 ± 4.66		
Chest X-ray	Normal		
SGOT (U/L)	23.73 ± 7.40		
SGPT (U/L)	23.86 ± 11.64		
Albumin/globulin ratio	1.19 ± 0.17		
Abdominal ultrasonography	Normal renal size and shape, 2 individuals with small kidney stone on the left-side (5-6 mm)		
Scintigraphy renal function	Left: 42.42 ± 2.82 Right: 43.11 ± 2.87		
Renal arteries on CT scan	Normal		
Creatinine (mg/dL)	0.85 ± 0.13	0.97 ± 0.16	0.045
eGFR (mL/min/1.73m^2^)	94.50 ± 5.39	71.04 ± 11.54	<0.001
Addis counts for erythrocytes	15/15 (100.0%) negative (<1000 erythrocytes/minute)		
Addis counts for leukocytes	15/15 (100.0%) negative (<2000 erythrocytes/minute)		
A/C ratio	13.3 (6.27 – 16.7)	12.2 (9 – 23.7)	0.322
24-hour proteinuria	15/15 (100.0%) negative		

## Discussion

This is the first study investigating the safety, clinical and laboratory characteristics of donors with thalassemia minor in living donor kidney transplantation. Donors with thalassemia minor are alive with overall good health after 2 (1-4) years follow-up. Both anemia condition and renal function of most participants were stable and well-managed.

Inherited diseases of hemoglobin are recognized as an increasing global health burden, of which thalassemia is one of the most common disorders [8]. To determine the eligibility for living donor kidney transplant, thalassemia is often classified into two groups, respectively transfusion-dependent and non-transfusion-dependent thalassemias. Since patients with transfusion-dependent thalassemia often suffer from iron overload and other complex complications and sequelae, only the group that does not require lifelong regular blood transfusions are acceptable for living donor kidney transplantation [9]. For living donor liver transplants, donors with thalassemia minor and those with correctable anemia are considered eligible [10].

The most prevalent gene mutation among thalassemia donors is DEL-SEA, which is consistent with several previous studies in the Asian population [5, 11]. These gene mutations are often associated with asymptomatic or mild anemia [5, 11], which explained the reason that the anemia condition among the donors in our study can only be detected via blood test. Thalassemia minor donors in our study who showed no clinical manifestations therefore can participate in living kidney donation if there are no other contraindications to kidney donation. Before kidney donation, there were 10 donors with mild anemia (Hb: 111.60 ± 8.71 g/L). Currently, there is no guideline that recommend the severity of anemia that is eligible for kidney transplant, we accepted donors with mild anemia with caution and actively treat and monitor the anemia condition pre- and post-surgery. After 1-5 years of follow-up, we found that nephrectomy did not affect the thalassemia disorder as the anemia of participants did not worsen among the donors.

Regarding renal function, before kidney donation, we examined several criteria on the donors, including creatinine, eGFR, proteinuria and also investigated specific function of each kidney individually with renal scintigraphy, which are all within acceptable range. It is proposed that high eGFR pre-nephrectomy is a protective factor against low post-donation eGFR [12], therefore we strictly followed the guidelines from Amsterdam Forum to only accept donors with eGFR > 80 mL/min/1.73m^2^ [13]. After 1-5 years, renal function of the donors were stable, the majority of participants have eGFR > 60 mL/min/1.73m^2^ and no eGFR of less than 30 mL/min/1.73m^2^ was seen. The decrease is acceptable considering a 50% reduction in renal mass due to nephrectomy and the age of our donors. Post-donation eGFR of the donors with thalassemia minor (71.04 ± 11.54 mL/min/1.73m^2^) is better than donors with hypertension (61.0 ± 2.0) reported by Textor et al. in a 282 days cohort [14] and we expected that the outcomes could be comparable to healthy donors (67.0 ± 14.0) after 5 years in a study conducted by Price et al. [15].

Specifically, Donors 2 and 13 had a drop in eGFR of >30% compared to the pre-donation eGFR and the resultant post-donation eGFR was <60 ml/min/1.73m^2^ after 20 months and 23 months of follow-up, respectively. This is not unexpected since a meta-analysis on healthy donors has reported a percentage of 12% donors would developed an eGFR < 60 ml/min/1.73m^2^ after 3-20 years [16]. Furthermore, both Donors 2 and 13 are > 50 year-old, which may be another factor. A cohort conducted by Lam et al. showed that after 2 years of follow-up, their healthy donors who aged 50 and above have a mean eGFR of 58.4 ± 9.8 ml/min/1.73m^2^ [17]. The eGFR is reported to be declined more rapidly in the elderly [17], but is likely to remain stable rather than progressively decline and thus still considered to be safe [18]. To ensure the safety of live kidney donation by the elderly, we recommend only accepting elderly who have a pre-operation eGFR > 90 ml/min/1.73m^2^ and who do not have hypertension or are overweight, which are known risk factors of developing chronic kidney diseases [17, 18]. Interestingly, we observed an increase of eGFR in Donor 1, which is can be caused by renal compensation [19].

Regarding two cases with increased A/C ratio values, Donor 10 and Donor 13 have the possibility of kidney damage occurred at 1 year after kidney transplant. The donors will need to be closely monitored the occurrence of any clinical manifestation and retest in future follow-up to assess whether this is transient or persistent proteinuria. If the proteinuria condition is persistent and progressing, we can confirm that this is kidney damage post-transplant. Although it is suggested that proteinuria is a common condition among living kidney donors which shall not worsen long-term renal function [19], this indicates the importance of carefully screening for kidney damage pre-transplant, and awareness should be raised on thalassemia donors with proteinuria, even when it is only mild albuminuria.

The major limitations of this study are its small sample size and short follow-up. The donors were followed during a median of 2 years (IQR 1 – 4 years) with a longest duration of up to 5 years. We believe these preliminary results should be reported to inform the short-term safety of thalassemia minor donors and highlights some criteria that need to be taken into account in these specific individuals, hopefully to assist and encourage evidence-based decision making in kidney transplants globally. Further long-term studies on thalassemia minor donors with larger sample size and control groups would be of great clinical significance.

## Conclusions

In conclusion, individuals with thalassemia minor who are non-transfusion-dependent, without anemia clinical manifestations and have no contraindications to kidney donation are safe to be donors in short-term. Estimated glomerular filtration rate of at least 80 mL/min/1.73m^2^ should be considered as an important criterion to avoid low post-donation eGFR, and awareness should be raised on thalassemia donors with even mild albuminuria. Nephrectomy does not worsen thalassemia condition.

## Supplementary Information

**Additional file 1.**

## Data Availability

All data generated or analysed during this study are included in this published article (and its supplementary information files).
