# Supplementary material for: Safety, clinical and laboratory characteristics of donors with thalassemia minor in living donor kidney transplant: a case series
Source: BMC Nephrol. 2021 Dec 1;22:397. doi: 10.1186/s12882-021-02609-2 (PMC8638172; doi:10.1186/s12882-021-02609-2)
Supplement: Supplementary file 1 — Additional file 1. [file 12882_2021_2609_MOESM1_ESM.docx]

Supplementary Table 1. Detailed clinical and laboratory characteristics of individuals

| Donor | Sex | YOB | Age | Thalas-semia | Gene mutation | Comorbidity | Relation-ship to patient | Trans-plant date | Last follow-up date | Follow-up period (months) | Post-surgery complications | Pre-op Hb (g/L) | Latest Hb (g/L) | Pre-op Hct (%) | Latest Hct (%) | Pre-op BP (mmHg) | Latest BP (mmHg) | Pre-op HR (bpm) | Latest HR (bpm) |
| --- | --- | --- | --- | --- | --- | --- | --- | --- | --- | --- | --- | --- | --- | --- | --- | --- | --- | --- | --- |
| Donor 1 | F | 1968 | 52 | Alpha | DEL-SEA | Kidney stone (L) 6mm | Mother | Jul-18 | Nov-20 | 28 |  | 97 | 94 | 37.8 | 35.2 | 120/60 | 120/70 | 84 | 70 |
| Donor 2 | F | 1957 | 63 | Alpha | DEL-SEA |  | Mother | Apr-19 | Dec-20 | 20 |  | 108 | 110 | 35.3 | 35.9 | 130/80 | 130/80 | 80 | 78 |
| Donor 3 | F | 1972 | 48 | Alpha | DEL-SEA |  | Mother | Aug-20 | Dec-20 | 4 |  | 91.6 | 116 | 28.7 | 34.5 | 120/70 | 120/70 | 74 | 70 |
| Donor 4 | F | 1975 | 46 | Alpha | DEL-SEA |  | Wife | Dec-19 | Apr-21 | 16 |  | 109 | 102 | 36.3 | 37.4 | 140/80 | 130/80 | 80 | 84 |
| Donor 5 | F | 1975 | 46 | Alpha | DEL-SEA |  | Sister | Oct-19 | Mar-21 | 17 |  | 123 | 132 | 38.9 | 41.9 | 105/56 | 110/70 | 70 | 74 |
| Donor 6 | F | 1955 | 66 | Alpha | Unknown |  | Mother | May-16 | Apr-21 | 60 |  | 90 | 100 | 36.1 | 33.8 | 120/70 | 110/70 | 84 | 74 |
| Donor 7 | F | 1961 | 60 | Alpha | DEL-SEA | Hypertension controlled with Concor 2.5mg/day | Mother | Jul-16 | Apr-21 | 58 |  | 108 | 114 | 37.2 | 36 | 140/80 | 130/70 | 88 | 88 |
| Donor 8 | M | 1950 | 71 | Hemoglobinose | Unknown |  | Father | Apr-17 | Apr-21 | 49 |  | 135 | 117 | 39.5 | 38.7 | 130/80 | 130/80 | 70 | 74 |
| Donor 9 | M | 1970 | 50 | Alpha | c-78A>G of HbB | Benign prostatic hyperplasia | Father | Apr-17 | May-20 | 38 |  | 140 | 140 | 45.2 | 44 | 110/70 | 110/70 | 70 | 74 |
| Donor 10 | F | 1959 | 61 | Alpha | DEL-SEA |  | Mother | Jun-19 | Nov-20 | 17 | Monitor kidney damage post-donation | 122 | 117 | 38 | 38.5 | 110/70 | 110/70 | 74 | 74 |
| Donor 11 | M | 1970 | 50 | Alpha | DEL-SEA | Kidney stone (L) 6mm | Father | Jun-18 | Nov-20 | 29 |  | 137 | 132 | 43.7 | 37.3 | 110/80 | 110/80 | 70 | 78 |
| Donor 12 | M | 1962 | 59 | Alpha | DEL-SEA |  | Father | Aug-17 | Apr-21 | 45 |  | 133 | 138 | 41.9 | 46.7 | 140/90 | 120/70 | 80 | 80 |
| Donor 13 | F | 1970 | 50 | Alpha | DEL-SEA | Kidney stone (L) 5mm | Mother | Dec-18 | Nov-20 | 23 | Monitor kidney damage post-donation | 132 | 113 | 41.1 | 37.8 | 130/80 | 130/80 | 84 | 80 |
| Donor 14 | F | 1962 | 59 | Beta | DEL-SEA |  | Mother | Jul-16 | Apr-21 | 58 |  | 117 | 112 | 38.9 | 36.2 | 110/70 | 120/70 | 78 | 88 |
| Donor 15 | F | 1962 | 58 | Alpha | DEL-SEA | Hypertension controlled with Tanatril 10mg/day, kidney stone (L) 5mm | Mother | May-19 | May-20 | 12 |  | 122 | 119 | 38.4 | 37.8 | 140/80 | 130/80 | 84 | 80 |

Supplementary Table 1 (continue).

| Patient | Pre-op A/C ratio (mg/g) | Latest A/C ratio (mg/g) | Pre-op Creati-nine (mg/dL) | Latest Creati-nine (mg/dL) | Pre-op eGFR (mL/min/  1.73m^2^) | Latest eGFR (mL/min/  1.73m^2^) | Latest Na  (mmol/  L) | Latest K (mmol/  L) | Latest Cl (mmol/  L) | Latest Ca (mmol/  L) | Latest urine pH | Latest urine sg | Latest urine glucose (mg/dL) | Latest urine protein (mg/dL) | Latest urine bilirubin (mg/dL) | Latest urine uro-bilinogen (mg/dL) | Latest urine ketone | Latest urine blood (RBC/  uL) | Latest urine leukocyte (WBC/  uL) | Latest urine nitrite |
| --- | --- | --- | --- | --- | --- | --- | --- | --- | --- | --- | --- | --- | --- | --- | --- | --- | --- | --- | --- | --- |
| Donor 1 | 5.56 | 6.5 | 0.82 | 0.67 | 92 | 101.06 | 139 | 3.8 | 106 | 2.2 | 6 | 1.008 | neg | neg | neg | norm | neg | neg | neg | neg |
| Donor 2 | 23.49 | 12 | 0.79 | 1.07 | 84 | 55.2 | 143 | 4.3 | 104 | 2.4 | 7 | 1.02 | neg | neg | neg | norm | neg | neg | neg | neg |
| Donor 3 | 17.6 | 25 | 0.82 | 0.96 | 99 | 69.93 | 137 | 3.6 | 102 | 2.4 | 7 | 1.003 | neg | neg | neg | norm | neg | neg | 125 | neg |
| Donor 4 | 16.4 |  | 0.62 | 0.98 | 100 | 69.18 | 136 | 3.8 | 105 | 2.4 | 5 | 1.025 | neg | neg | neg | norm | neg | neg | neg | neg |
| Donor 5 | 3.37 | 6.25 | 0.69 | 0.82 | 105 | 85.81 | 136 | 3.5 | 104 | 2.4 | 6 | 1.02 | neg | neg | neg | norm | neg | neg | neg | neg |
| Donor 6 | 56 | 9 | 0.81 | 0.91 | 96 | 72.54 | 136 | 4 | 107 | 2.3 | 5.5 | 1.015 | neg | neg | neg | norm | neg | 10 | neg | neg |
| Donor 7 | 17 | 10.1 | 0.96 | 0.88 | 85 | 71.41 | 137 | 3.9 | 102 | 2.4 | 7 | 1.009 | neg | neg | neg | norm | neg | neg | neg | neg |
| Donor 8 | 6 | 23.4 | 1.02 | 1.08 | 92 | 68.69 | 137 | 4.4 | 102 | 2.3 | 5.5 | 1.015 | neg | neg | neg | norm | neg | neg | neg | neg |
| Donor 9 | 7.34 | 2.57 | 1.01 | 1.23 | 99 | 68.02 | 141 | 4 | 108 | 2 | 5 | 1.026 | neg | neg | neg | norm | neg | neg | neg | neg |
| Donor 10 | 8.05 | 173 | 0.68 | 0.99 | 95 | 61.5 | 134 | 3.5 | 102 | 2.3 | 5.5 | 1.01 | neg | 15 | neg | norm | neg | neg | 15 | neg |
| Donor 11 | 6.3 |  | 0.98 |  | 93 |  |  |  |  |  |  |  |  |  |  |  |  |  |  |  |
| Donor 12 | 13.3 | 23.7 | 1.1 | 1.24 | 95 | 63.23 | 136 | 4.2 | 103 | 2.3 | 7 | 1.011 | neg | neg | neg | norm | neg | 80 | 70 | neg |
| Donor 13 | 14.7 | 34.2 | 0.9 | 1.1 | 92 | 58.49 | 135 | 4 | 100 | 2.5 | 5 | 1.015 | neg | neg | neg | norm | neg | neg | neg | neg |
| Donor 14 | 16 | 23.4 | 0.86 | 0.84 | 95 | 76.08 | 141 | 3.7 | 106 | 2.4 | 6 | 1.021 | neg | neg | neg | 1 | neg | neg | neg | neg |
| Donor 15 | 6.25 | 12.2 | 0.8 | 0.87 | 96 | 73.43 | 140 | 4 | 105 | 2.1 | 7 | 1.008 | neg | neg | neg | norm | neg | neg | neg | neg |
